# Creating safe spaces: fostering inclusion in animal science classrooms through DEI initiatives

**DOI:** 10.1093/af/vfaf029

**Published:** 2025-10-29

**Authors:** Nicole M Tillquist

**Affiliations:** Department of Animal Science, University of Connecticut, Storrs, CT 06269

**Keywords:** animal science, diversity, LGBTQ+, inclusion, pedagogy

**Implications**

- A student’s sense of safety and belonging in academia can be fostered through inclusive pedagogy and other diversity, equity, and inclusion initiatives.
- The needs of students who identify within the lesbian, gay, bisexual, transgender, queer plus community have historically been overlooked in animal sciences.
- Animal science faculty who are committed to lesbian, gay, bisexual, transgender, queer plus inclusivity can consider modeling the use of pronouns, avoid assumptions about student gender identities, facilitate community involvement, and actively seek diversity, equity, and inclusion trainings.

## Introduction

Diversity, equity, and inclusion (DEI) in higher education is critical for the success of not only those of marginalized identities but all students (Conron et al., 2022), and commitment to DEI is an essential responsibility of educators across all disciplines (Elliott-Engel et al., 2019; Bullock et al., 2021). The absence of inclusive pedagogy actively creates isolating learning environments. Ideally, students should be learning in spaces where they feel comfortable expressing their opinions and asking questions, regardless of their cultural or educational backgrounds, gender identity, age, sexual orientation, religion, race, or political views. It is important that animal science and agricultural-focused programs make intentional efforts to prioritize DEI, and increased attention on the experiences of students who identify within the lesbian, gay, bisexual, transgender, queer plus (LGBTQ+) community is particularly needed within these departments. The specific needs of these students remain underrepresented in conversations about DEI promotion (Elliott-Engel et al., 2019). This, in part, is due to a lack of available sexual orientation and gender identity/expression (SOGIE) data and qualitative research based on the lived experiences of students from underrepresented groups enrolled in agricultural degree programs (Bullock et al., 2021). This presents a gap that hinders our ability to truly understand and address the specific concerns and lived experiences of these students. The Williams Institute (https://williamsinstitute.law.ucla.edu) at the University of California, Los Angeles, is a publicly available resource of SOGIE data. In 2022, the experiences of LGBTQ+ identifying students in 4-year degree programs and graduate programs across the United States were evaluated, and themes of lack of belonging or hiding, mental health struggles, and reports of bullying, harassment, and assault were reported (Conron et al., 2022). Despite originating outside of agricultural degree programs, these data hold direct relevance for LGBTQ+ students and those whose identities intersect with other marginalized groups, such as LGBTQ+ people of color.

**Figure 1.**
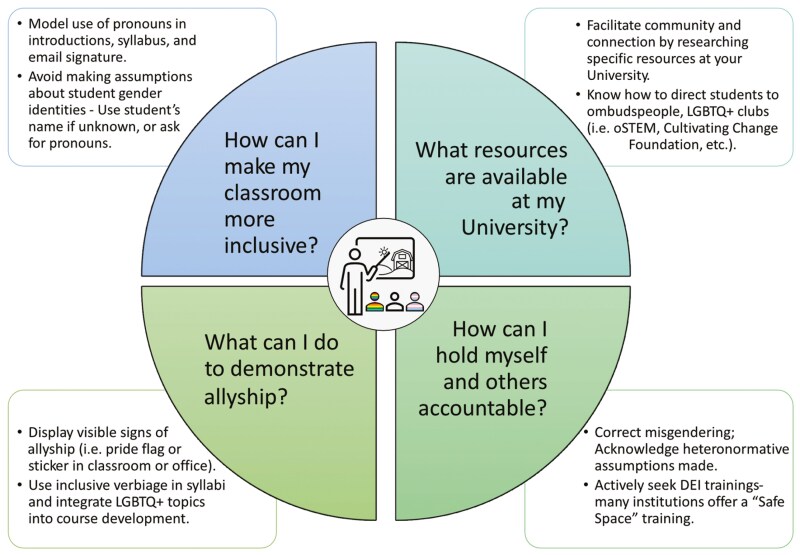
Considerations for faculty committed to LGBTQ + inclusivity

Inclusion efforts specific to the needs of students who identify within the LGBTQ+ community have historically been overlooked and understudied in agricultural education (Murray et al., 2020). Recent work specific to the Animal Science degree program at Iowa State University explored the perspectives of undergraduates in the department on DEI and belonging and found that female students identifying as Hispanic report feelings of lack of inclusion in classes, with peers, and with faculty (Carlson et al., 2024). Demographics related to sexual orientation and non-cisgender identity were not collected in this survey, highlighting a lack of acknowledgement of the LGBTQ+ community when discussing DEI efforts, and a missed opportunity to identify feelings of belonging in students who have intersecting marginalized identities. Animal and agricultural science programs would benefit from SOGIE data to understand the specific needs of their student population, though there are a number of immediate action steps that can be taken by faculty members to support students who identify within the LGBTQ+ community (Figure 1).

Here, I aim to explore how small, yet significant, shifts in classroom practices and culture can make a meaningful impact on LGBTQ+ students. In creating an inclusive environment through DEI efforts in academia, we are not only supporting the student body, but also improving the overall culture of the institution (Elliott-Engel et al., 2019). Though this paper focuses on the unique needs of one marginalized community, it is imperative that faculty members are committed to understanding the impacts of racism, implicit bias, and microaggressions on students from all underrepresented groups, and that we continue to identify actionable ways to create change.

## Creating Foundational Respect

The social culture at a University directly impacts student identity development (Elliott-Engel et al., 2019), and faculty members have a vital role in contributing to that social culture. To ensure that all students feel safe, it is important to establish a foundation of mutual respect. Regarding the needs of LGBTQ+ students, this can include asking for and using students’ pronouns and preferred names. A faculty member striving to create an inclusive space for LGBTQ+ students can also model use of pronouns in classroom introductions (i.e., “My name is Dr. Smith, my pronouns are she/her”), and include their pronouns on syllabi and in their email signature. Explicitly denoting expectations for respectful communication and acceptance amongst peers in a syllabus or on a departmental website can help to convey a commitment to valuing individual differences. These actions will help to establish safety and demonstrate to students that you are making a tangible effort to create an inclusive environment.

## Signaling Allyship

Allyship (explicit support for LGBTQ+ individuals) has been reported to be of utmost importance to LGBTQ+ students (Elliott-Engel et al., 2019). Allyship can be shown via visible indicators such as pronoun pins and LGBTQ+ resources displayed in the classroom or office, and can immediately create a sense of safety and belonging. Allyship can also be demonstrated by acknowledging diverse backgrounds and experiences, and explicitly incorporating related content into course material, such as including LGBTQ+ topics in the curriculum and related discussion (i.e., avoiding heteronormative definitions of family), or inviting speakers who identify as LGBTQ+ to provide representation of successful individuals in the field.

## Facilitating Community and Connection

Being a part of an unwelcoming community during one’s education can deter student interest in pursuing an agricultural career, and not all spaces on a University campus are regarded as “safe spaces” (Elliott-Engel et al., 2019). Therefore, it is imperative that we create more openly safe environments to ensure student retention, engagement, and sense of belonging. Faculty members can help to foster an inviting and accepting community for LGBTQ+ individuals by being aware of what resources exist at their institution that provide support, connection, and validation. Faculty can also involve themselves in initiatives on campus, such as community building at LGBTQ+ specific clubs (i.e., oSTEM, Cultivating Change Foundation; Figure 1) or participation in events that support queer youth (i.e., Queer Science).

## Continuous Education and Accountability

Seeking continuous education specific to understanding and facilitating DEI initiatives is another important step that faculty can take to support students from marginalized backgrounds. While these topics may be included in some basic annual training requirements, faculty can increase their awareness and ability to support LGBTQ+ students by accessing available resources such as trainings on bystander intervention, implicit bias, etc. Additionally, it is essential that faculty demonstrate a commitment to holding themselves and others accountable when bias and discrimination inevitably impact safety within the classroom or department community. Correcting misgendering of a student, condemning any use of derogatory or dismissive language, and offering individual support to students who have experienced micro- or macroaggressions all go a long way in ensuring our communities are truly safe places for all learners.

## Conclusion

A critical need exists for animal science educators to actively engage in creating inclusive learning environments. As researchers in our field, we must prioritize the collection of SOGIE data on LGBTQ+ youths to better understand the needs of all students. Increased research on LGBTQ+ youth involved in agricultural education will impact hundreds of thousands of students (Murray et al., 2020). It is important that educators in higher education possess the tools needed to create safe, inclusive spaces for LGBTQ+ students to thrive upon entering a college classroom. Here, I have highlighted several steps that animal science/agricultural science faculty members can take to contribute to LGBTQ+ students feeling welcomed. It is my hope that readers can benefit from the information provided and act today to create change within their institution.

## About the Author

Nicole Tillquist, PhD (she/her), is a postdoctoral research associate at the University of Connecticut. She is a proud, queer woman in animal science and is actively engaged in efforts to educate faculty and graduate students on inclusive pedagogy, mentor queer youths in her community, and advocate for the need for more data on sexual orientation and gender identity/expression in animal sciences.
